# Identifying key domains of health-related quality of life for patients with Chronic Obstructive Pulmonary Disease: the patient perspective

**DOI:** 10.1186/s12955-014-0106-3

**Published:** 2014-07-09

**Authors:** Muirne CS Paap, Christina Bode, Lonneke IM Lenferink, Lianne C Groen, Caroline B Terwee, Sara Ahmed, Owis Eilayyan, Job van der Palen

**Affiliations:** 1 Department of Research Methodology, Measurement, and Data-Analysis, Behavioral Sciences, University of Twente, Enschede, 7500 AE, The Netherlands; 2 Department of Psychology, Health & Technology, Behavioral Sciences, University of Twente, Enschede, The Netherlands; 3 University of Twente, Enschede, The Netherlands; 4 Current affiliation: Department of Clinical Psychology, Behavioural and Social Sciences, University of Groningen, Groningen, The Netherlands; 5 Department of Epidemiology and Biostatistics, The EMGO Institute for Health and Care Research, VU University Medical Center, Amsterdam, The Netherlands; 6 School of Physical and Occupational Therapy, Faculty of Medicine, McGill University; with concordant appointments at McGill University Health Center’s Division of Clinical Epidemiology; and, the Centre de recherche interdisciplinaire en réadaptation, Constance Lethbridge Rehabilitation Center, Montréal, Québec, Canada; 7 Faculty of Medicine, School of Physical Therapy, McGill University, Montreal, QC, Canada; 8 Medical School Twente, Medisch Spectrum Twente, Enschede, The Netherlands

**Keywords:** HRQoL, COPD, Physical functioning, Physical health, Social health, Interview study, Qualitative research, PROMIS

## Abstract

**Background:**

Numerous instruments are available to measure health-related quality of life (HRQoL) in patients with Chronic Obstructive Pulmonary Disease (COPD), covering a wide array of domains ranging from symptoms such as dyspnea, cough and wheezing, to social and emotional functioning. Currently no information or guide is available yet to aid the selection of domains for a particular study or disease population. The aim of this paper is to identify which domains of HRQoL are most important with respect to COPD, from the patient perspective.

**Methods:**

Twenty-one Dutch patients with COPD were asked to describe important domains impacted by COPD freely; second, they were presented with cues (domains from the Patient-Reported Outcomes Measurement Information System (PROMIS) framework) and were asked to select the domains that were most relevant to them. During the interview, the patients were asked to indicate in which way the selected domains impact their lives. Both the answers to the open question, and the patient statements motivating nomination of PROMIS domains were coded into themes.

**Results:**

The most relevant (sub)domains of HRQoL for patients with COPD were: physical health (fatigue, physical functioning), social health (instrumental support, ability to participate in social roles and activities, companionship, and emotional support), and coping with COPD.

**Conclusion:**

We identified which domains of HRQoL are most important to patients with COPD. One of these (coping with COPD) is not explicitly covered by PROMIS, or by traditional questionnaires that are used to measure HRQoL in COPD.

## Background

Chronic Obstructive Pulmonary Disease (COPD) is one of the leading causes for mortality worldwide [1]. COPD is characterized by dyspnea, chronic cough, sputum production, a decreased exercise performance and reduced physical activity level [2],[3]. COPD cannot be cured; the main goal of COPD treatments is managing symptoms and their effect on the patient’s health-related quality of life (HRQoL) [3]. Consequently, HRQoL has become an important outcome measure in COPD research and clinical care.

HRQoL encompasses the physical, functional, emotional, and social well-being of the patient [4]. HRQoL is a highly subjective experience by definition and therefore measured by self-report questionnaires (patient reported outcome measures; PROMs). Many questionnaires are available to measure HRQoL in patients with COPD, covering a wide array of domains ranging from symptoms such as dyspnea, cough and wheezing, to social and emotional functioning (see [5] for an overview), and it can be a challenge to choose the appropriate questionnaire. Aspects to consider include domain coverage, test length (e.g. [6],[7]), recall period (for example, last year or last few weeks), the interpretation of (change in) scores, psychometric properties (e.g. [8]), and whether it is generic or disease-specific, among other things [9]. Since both generic and disease-specific instruments have desirable properties, several researchers advocate using both types of instruments to assess HRQoL in patients with COPD [10],[11]. Generic instruments can be used with any (patient) population, facilitating direct comparison among populations, including the general population. Disease-specific instruments are developed for a specific patient population, which may increase the sensitivity to measure intra-individual change after an intervention. However, clinicians tend to prefer short instruments for use in clinical practice, so the test battery should not be too lengthy. Unfortunately, short instruments often fail to provide a sufficiently detailed picture of the most relevant aspects of a patient’s HRQoL.

In recent years, Computerized Adaptive Testing (CAT) [12] based on Item Response Theory (IRT) [13] has been put forward as a possible solution to the dilemma. A CAT is a digital questionnaire tailored to the individual patient, resulting in each item (question) contributing valuable information, while maintaining comparability across patients. Item selection in a CAT is dependent on a patient’s response to previous items. In this way the latent trait estimate is continuously adjusted, until a specific level of measurement precision (reliability) is reached. In recent years, a large number of IRT-calibrated item banks have been developed to measure domains of HRQoL. This initiative is referred to as “PROMIS” (Patient Reported Outcomes Measurement Information System) [14], and resulted in item banks covering physical, mental, and social health. Both CATs and short forms have been developed using the item banks [15]. The PROMIS banks are generic, and have been calibrated using a large sample from the general population as well as subsamples of numerous patient groups, including COPD. However, it is still up to the user to decide which domains to select.

Since HRQoL is a highly subjective experience, it is not only paramount to use PROMs to measure it, but also to involve patients in the development and selection process of PROMs. It has been repeatedly shown that taking patient perspective into account during PROM development allows for the identification of problems with item content and coverage as well as problems with domain coverage and/or poor operationalization (e.g., [16]-[27]). These findings have inspired several guidelines concerning the development of PROMs. The European Organisation for Research and Treatment of Cancer (EORTQ) even proposes that patient perspective should be *leading* when “…compiling an exhaustive list of relevant QoL issues that cover the domain(s) of interest” [28]. Within the PROMIS framework, patient perspective has played an important role in item bank development [29]-[32]. Focus-groups were used to evaluate item content, coverage and wording, as well as the content validity of the item banks by evaluating the fit between the domain map that was drafted by a panel of experts, and concepts that focus-group participants identified as important aspects of HRQoL [33]-[36]. It should be noted that in this set-up, feedback from experts was used to conceptualize a domain-map, and subsequently input from patients (responding to open questions) was used to verify the domain map. Patients were not explicitly asked to comment on the domain-map, or to choose domains themselves.

Currently, there is no consensus as to which instruments are most appropriate to assess HRQoL in COPD patients. However, it has been argued that combining generic and disease-specific measures may provide the most useful estimations. PROMIS is probably the best generic instrument that is currently available to measure HRQoL; it consists of a wide range of generic item banks that have been developed with a rigorous and sound methodology. However, no core sets or recommendations are available to guide the selection process of PROMIS item banks for use with COPD patients (or any disease group for that matter); nor is it clear whether all domains relevant for COPD are covered by current PROMIS item banks. In line with aforementioned guidelines and published studies, we agree the patient perspective should play an important role in domain selection and assessment of domain coverage. The aim of this paper is to identify which domains of HRQoL are most important from the COPD patient’s perspective, and why. We will do this in two steps: first patients are asked to describe important domains freely; second, they are presented with PROMIS domains and are asked to select those most relevant to them. The patient is invited to explain the motives behind their choice, providing insight in the way they conceptualize and experience HRQoL. The results of this study can be used to inform instrument selection and development. This study is the first of an international collaboration (the Netherlands and Canada) that involve two research groups following the same steps (Additional file 1) to create a CAT to measure HRQoL in COPD patients.

## Methods

### Context of the study

This exploratory study was initiated in order to establish which domains of HRQoL are most relevant to patients with COPD. MP and JP acquired funding from the Dutch Lung Foundation in order to develop a new instrument to measure HRQoL in patients with COPD. However, at present no guidelines are available that can aid an interested party to select appropriate domains for their purpose (e.g., instrument development, clinical trial, clinical evaluation); this applies to PROMIS domains as well as legacy instruments. Therefore, MP and JP decided to start their project by conducting an interview study. CB was involved as an expert in qualitative research and patient perspective, and CT was involved because of her affiliation with the PROMIS initiative, as well as her methodological expertise. SA and OE were involved since they had the same goals as MP and JP; it was decided that an international collaboration would increase the overall value and impact of the project. Due to time constraints, it was decided that the interview study would be executed in the Netherlands first. The drafting of the framework was a joint effort. The interview scheme used in this study was translated into English and will be used in a future study to validate the findings of the current study in Canada.

### Patients

The target population consisted of COPD patients seeking treatment. We engaged two pulmonary clinics that assisted in recruiting patients: Medisch Spectrum Twente hospital, Enschede (the Netherlands) and the Sint Franciscus Gasthuis hospital, Rotterdam (the Netherlands). The patients were invited to participate in the interview study by their pulmonologists. The only inclusion criterion was a diagnosis of COPD. If the patients agreed to participate, they were taken to a quiet hospital room where the interviewer explained the study in more detail, and presented the patients with an informed consent form before starting the interview. Consecutive patients who had appointments with their pulmonologists in March and April 2013 were considered for inclusion. Purposive sampling was used, aimed at maximum obtainable variation with regard to patient characteristics, including gender, age and GOLD stage (I&II and III&IV). Inclusion stopped when saturation was reached.

### Interview

The interview was semi-structured (see Additional file 2 for the interview scheme). The focus was assessing how COPD impacts HRQoL from the patient’s perspective. The interview scheme consisted of two parts: an open question, and a card sorting task. First, patients were asked: “In what way does your COPD influence your quality of life?” Next, participants were presented with 16 PROMIS domains [37] printed on separate cards, along with three randomly chosen example items from that domain (see Additional file 3). The patients were invited to select and rank the five domains that were the most relevant to them with regards to their COPD. Patients were asked to think aloud while making this selection, and elaborate on their choices. Each interview was recorded and transcribed verbatim.

At the time of the interview, Dutch translations of 17 PROMIS domains were available. We chose not to use the domain “global health” since we wanted to know what specific domains of HRQoL were most relevant from the patient’s perspective. We felt that ranking all 16 domains would be too challenging, so we decided to divide the task in two parts: first the most important domains were chosen, and then these were ranked. Previous research has shown that rating and ranking five domains is a doable task [38], so patients were asked to select and rank five domains.

### Ethics

The ethical review board of the University of Twente approved the study. All patients gave informed consent. This study did not need approval of the Medical Ethical Review Board, according to European regulations.

### Data analysis

The interviews were held and transcribed by a trained interviewer (LG). Patients’ statements were coded and interpreted by hand, and the number of times a domain was chosen was counted. After choosing the domains, the patients were asked to rank order these domains from most relevant to least relevant, but not all patients followed the instructions. Some patients chose fewer or more than five domains, and some said they could not rank them because they felt all were equally (un)important. We therefore decided to not include the rank orders of the domains in the analysis.

Coding was performed in several steps by two raters (LG and LL). The coding process was supervised by CB and MP. LG and LL first coded the interviews on their own, after which several consensus meetings took place. In challenging cases, MP was involved in the consensus process. The open question and PROMIS card sorting task were analysed separately, yet the same coding procedure was used. Prior to coding, irrelevant interview passages (spontaneous statements that do not concern the research question) were removed. We used two types of coding strategies described in grounded theory research: open and axial coding [39]. When using open coding, the data are broken down into units (events, actions, interactions, emotions) that are assigned conceptual labels, so they can be grouped together into themes. Meaningful units constitute parts of a sentence, a whole sentence, or a passage of text that pertains to the same topic. A meaningful unit has to be long enough (contain enough information) to allow meaningful interpretation with respect to the research question and the theme with which it is associated. When using axial coding, units are related to themes and subthemes. In our study, this process was facilitated by what we refer to as interpretations of meaningful units (Table 1). For example, the unit “When I have to walk up and down the stairs, I move up the stairs in a sitting position and then I’m very short of breath” was interpreted in the following way: “Going up the stairs is a difficult and laborious exercise, which causes shortness of breath.” This unit was subsequently placed in the sub-theme “Light physical activity causes physical complaints”, which was associated with the main theme “Physical health”. The main themes *physical health* and *autonomy* which emerged from the open question, and the PROMIS domains *fatigue* and *ability to participate in social roles and activities* were chosen to illustrate the coding process (Tables 1 and 2, respectively).

**Table 1:** Examples showing how units were interpreted and coded into themes for answers given to the open question

**Main theme**	**Sub-theme**	**Selected unit**	**Interpretation**
Physical health	Light physical activity causes physical complaints	“When I have to walk up and down the stairs, I move up the stairs in a sitting position and then I’m very short of breath.”	Going up the stairs is a difficult and laborious exercise, which causes shortness of breath.
Physical health	Heavy physical activity causes physical symptoms	“Especially, during heavy physical exertions, you feel some pain at times, and you experience some shortness of breath.”	Heavy physical exertion triggers pain and shortness of breath.
Physical health	Stamina	“My stamina is, of course, somewhat lower than others. I think that is actually the most important point.”	Stamina is lower in comparison to others.
Autonomy	Asking for help	“Now I have to ask others can you please help me? Mind you, I haven’t done it yet, but that’s very hard.”	It is difficult to ask others for help.
Autonomy	Independence	“Then I get irritable, because I like to do everything myself, and I’m not able to do that.”	Loss of independence causes anger.

**Table 2:** Examples showing how units were interpreted and coded into themes for two PROMIS domains

**Selected domain**	**Main theme**	**Sub theme**	**Selected unit**	**Interpretation**
Fatigue	Coping with fatigue	Rest	“When I’m at home and I’m tired, I really can’t be bothered with anything, the whole world may be turned upside down for all I care, all I want is to lie down.”	Lying down as a reaction to fatigue.
Fatigue	Effects of fatigue	Downward performance spiral of fatigue: fatigue leads to activity restrictions. Activities trigger breathing difficulties.	“Yes, you get tired more quickly. You’ve also got the feeling that if you want to do something…then you ask yourself right away should I do that or how should I go about it so that I won’t suffer breathing problems.”	Activity limitations due to fatigue, and activities trigger breathing problems.
Fatigue	Determinants of fatigue	Combination of work and medicines lead to fatigue.	“I’m always tired anyway, if I didn’t have to go to work I would be tired all the same, you actually get tired because of the medicines you have to take during the day. One medicine has to widen the blood vessels, the other has to narrow them again …it’s a combination of (the aforementioned).”	Combination of work and medicines lead to severe fatigue.
Ability to participate in social roles and activities	Determinants of ability to participate in social roles and activities	Mobility restrictions	“Because of your shortness of breath you are restricted in your mobility and therefore also in your social roles. A while ago, I was supposed to attend a funeral at XX…that’s a problem.”	Symptoms cause mobility restrictions as a result of which social contacts are not possible which in turn leads to limitation in social roles.
Ability to participate in social roles and activities	Hobby/Leisure time	Limitation in social role at sports club	“I used to be the leader of a football team, and I could run as fast as the boys, but those days are over.”	Quit participation in social role and sports activity
Ability to participate in social roles and activities	Hobby/Leisure time	Limitation during sports activity	“You have to limit it somewhat, don’t you?	Limitation in degree of physical activity during sports activity
			When I go swimming, for instance, I bring two people; well, those two are able to swim very well, when they have reached the other side of the pool for the second time, I have yet to reach the other side for the first time.”	

## Results

Twenty-one COPD patients (13 men; mean age 66.1 (SD = 9.3) years) were included; 24% of them were recruited in Rotterdam; 19% were inpatients; 9.5% had GOLD stage I, 38% GOLD stage II, 43% GOLD stage III, and 9.5% GOLD stage IV. The face-to-face interviews took 15–30 minutes. See Table 3 for descriptive characteristics for each patient.

**Table 3:** Patient characteristics

**ID**	**Gender**	**Age**	**GOLD stage**	**Location**	**Outpatient/inpatient**	**Comorbidity**
1	M	58	III	R	Outpatient	Yes
2	M	78	III	R	Outpatient	Yes
3	M	67	II	R	Outpatient	Yes
4	M	72	III	R	Outpatient	Yes
5	M	76	IV	R	Outpatient	Yes
6	F	66	III	E	Outpatient	No
7	M	68	III	E	Inpatient	No
8	M	52	I	E	Outpatient	Yes
9	M	63	III	E	Outpatient	Yes
10	M	52	II	E	Outpatient	Yes
11	M	54	II	E	Outpatient	Yes
12	F	58	II	E	Outpatient	Yes
13	F	69	II	E	Outpatient	No
14	F	67	III	E	Outpatient	Yes
15	F	81	II	E	Outpatient	Yes
16	F	62	IV	E	Outpatient	Yes
17	M	73	II	E	Outpatient	Yes
18	F	51	II	E	Outpatient	Yes
19	M	84	I	E	Inpatient	Yes
20	M	70	III	E	Inpatient	Yes
21	F	67	III	E	Inpatient	No

Location: R = Rotterdam, E = Enschede.

### Open question: “In what way does your COPD influence your quality of life?”

Table 4 presents the main themes that emerged from the analysis of the open question, along with sub-themes. Below, all main themes that emerged from the answers given by more than one patient are briefly discussed; for some themes, patient quotes are given.

Of the ten main themes, two were by far the most popular: physical health and coping with COPD-related complaints. Patients described that light physical activities, such as walking, climbing the stairs and doing household chores, trigger physical complaints. Decline of stamina, restrictions in general physical activity and invalidity were the next most common subthemes of physical health (see Table 1 for examples). Some patients indicated that the impact of their COPD on their physical activity fluctuated.

“There is a tram stop nearby… walking that distance…sometimes I can manage it, sometimes I can’t.” (ID 1)

Used coping strategies were: doing only as much as they could or trying to avoid activities that could lead to dyspnoea, and performing short bouts of light to moderate intensity activities in order to maintain a balance between rest and activity.

“You do your household chores as best as you can, but you also take a lot of rest breaks. In that way you’re able to cope with it.” (ID 6)

Some patients experienced limitations regarding hobbies and leisure time. Autonomy was mentioned several times; patients reported that it is difficult and frustrating to be dependent upon others (see Table 1 for examples). For some patients, their social lives changed negatively due to COPD. Anxiety or fear of suffocation was also experienced by some.

“And then I was at home alone and then I screamed for help: I thought I was going to suffocate. The feeling just wouldn’t pass.” (ID 5)

**Table 4:** Overview of main themes and sub-themes connected to the open question

**Main themes (number of occurrences)**	**Sub-themes (number of occurrences)**
**Physical health (22)**	Light physical activity triggers physical complaints (6)
	Stamina (4)
	Fluctuating character of COPD (4)
	Restriction in general physical activity (3)
	Invalidity (2)
	Restriction in physical functioning due to physical symptoms (1)
	Heavy physical activity causes physical complaints (1)
	Mobility (1)
**Coping with COPD-related complaints/restrictions (9)**	Avoiding activities (3)
	Balance between rest and activity (3)
	Performing physical activities in order to avoid sitting at home (1)
	Adapting activities (1)
	Professional help for anxiety (1)
**Hobby/Leisure time (3)**	Going out (1)
	Holidays (1)
	Sport (1)
**Autonomy (2)**	Asking for help (1)
	Independence (1)
**Anxiety (2)**	Fear of being alone (1)
	Fear of suffocating (1)
**Social life (2)**	Homebound (1)
	Offering help to others (1)
**Additional factors affecting symptoms (1)**	Weather conditions (1)
**Fatigue (1)**	Tired (1)
**Anger (1)**	Frustration (1)
**Work (1)**	Losing one’s job (1)

Examples of light physical activity are walking up and down the stairs. Examples of heavy physical activity are heavy lifting and running. General physical activity when the respondent did not give details.

### Selecting the most relevant PROMIS domains

**Figure 1:**
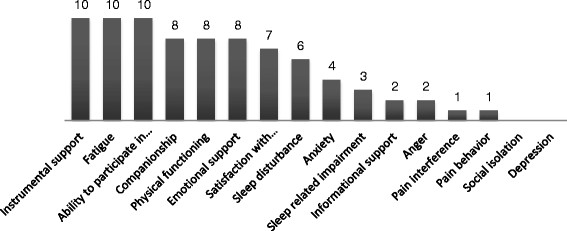
**Number of times PROMIS-domains were selected.***Note*. Ability to part. in soc. roles and act = ability to participate in social roles and activities; Satisfaction with part. in soc. roles and act. = satisfaction with participation in social roles and activities. The patients were instructed to select the five most relevant domains, but twelve patients chose fewer than five domains (four domains n=5, three domains n=3, zero domains n=4) and four patients chose more than five domains (six domains n=3, eight domains n=2).

The card sorting method produced results on the relative importance of the PROMIS domains (Figure 1). The statements that the patients gave when motivating their choice of domains were coded into themes. In Table 5, all main themes are listed by PROMIS domain.

**Table 5:** Overview of main themes derived from patient statements associated with the selected PROMIS domain

**Selected domain**	**Main themes**	N*
**Instrumental support**	Description of instrumental support	11
	Autonomy	2
	Coping with instrumental support	2
	Level of importance of instrumental support	2
**Fatigue**	Description of fatigue	6
	Coping with fatigue	4
	Determinants of fatigue	3
	Consequences of fatigue	2
	Physical activity	1
	Comorbidity leads to misconception	1
	Mobility	1
**Ability to participate in social roles and activities**	Hobby/Leisure time	6
	Coping with limitations in the ability to participate in social roles and activities	3
	Determinants of the ability to participate in social roles and activities	2
	Restrictions in physical activities	1
	Description of social roles and activities	1
	Level of importance of social roles and activities	1
**Companionship**	Description social contacts	10
	Pointing out positive results of social contacts	3
	Importance of social contacts	3
	Description social activities	1
	Level of satisfaction with company	1
	Pointing out negative results of social contacts	1
	Life and death	1
**Physical function**	Experiencing limitation or complaints during physical exertion	8
	Autonomy	2
	Additional factors affecting physical complaints	1
	Fatigue	1
	Level of satisfaction with physical function	1
**Emotional support**	Description of emotional support	8
	Level of importance of emotional support	2
	Pointing out positive results of emotional support	2
**Sleep disturbance**	Description of sleep disturbance	5
	Determinants of fatigue	1
	Consequences of stress	1
**Sleep related impairment**	Coping with problems caused by sleep disturbances	2
	Description of problems caused by sleep disturbances	1
	Comorbidity leads to misconception	1
**Satisfaction with participation in social roles and activities**	Autonomy	8
	Social life	1
	Description of attitude to life	1
	Determinants of fatigue	1
	Consequences of fatigue	1
	Anger	1
	Level of importance of being independent from others	1
**Informational support**	Description of informational support	3
**Anxiety**	Description of anxiety	4
	Consequences of physical complaints	1
**Pain interference**	Description of pain	1
	Description of limitations due to pain	1
**Pain behaviour**	Comorbidity leads to misconception	1
**Anger**	Determinants of anger	2
	Coping with anger	1
	Importance of coping with anger	1
	Description of anger	1
	Autonomy	1

*N = number of occurences.

For each selected PROMIS-domain the units were interpreted and coded into main and sub themes. Only the main themes are presented in this table.

The three most frequently chosen domains were *instrumental support*, *fatigue* and *ability to participate in social roles and activities. Instrumental support* was relevant for patients because they need help or assistance due to their physical constraints.

“Luckily, I have a domestic help, and if I hadn’t had that kind of help nothing would have gotten done…” (ID16)

In contrast, some patients indicated that they had trouble accepting help from others, and had to employ coping strategies to deal with this change in dependency.

Patients described *fatigue* as highly bothersome in various ways; a chronic lack of energy, feeling already totally exhausted when waking up or experiencing feelings of weariness, etc. The most frequently mentioned way to cope with fatigue was to lie down and rest. Patients were not always very clear on determinants of fatigue: it could occur without any reason, or result from physical activity or use of medication. See Table 2 for quotes. Patients associated *ability to participate in social roles and activities* primarily with hobbies and leisure time. They experienced restrictions in physical activities during holidays; for example, climbing stairs of a tower or hiking. Some described adjusting the intensity of sports activities and avoiding social activities. See Table 2 for more examples.

The next three most frequently chosen domains were *companionship*, *physical function* and *emotional support. Companionship* was described in terms of how content patients were with the companionship of others. Most statements consisted of summing up the social contacts, e.g. children, partner and grandchildren. *Physical function* was described as being restricted in daily life tasks, such as dressing, climbing stairs and vacuum cleaning. A few patients described themselves as physically disabled. Autonomy was another reported main theme.

“The feeling of powerlessness, that for everything you have always to rely on others to help you, that is annoying.” (ID 5)

*Emotional support* was relevant because patients have the need to talk to someone to be comforted, to vent their feelings, to discuss problems or ask for advice. Partners and children are the most favoured persons patients confide in. Some stated that talking to someone is ‘healing’, it prevents emotional problems from getting worse and it improves mood.

### Additional notable results

Not one patient explicitly chose the domain *depression*, even though this theme emerged several times during the interviews in connection with other domains or themes. Feelings of depression were associated with fatigue, autonomy and acute exacerbations of COPD.

“And fatigue, sometimes I’m exhausted and I feel depressed and I think heck, why can’t I do that, but there are also moments when I like it and don’t at all resent it. It’s so difficult.” (ID 5)

A few patients expressed fear of suffocating associated with respiratory distress, which lead to thoughts of wanting to die or not minding to die.

“When it is so bad and stays so bad then I just hope it will be over.” (ID 7)

Strikingly, the topic of autonomy was frequently mentioned in conjunction with various domains (see Table 5). Patients mentioned having trouble accepting help from others or that they avoid asking help from others. They get frustrated, angry or depressed when they are unable to do the things they want (independently). Losing one’s autonomy is a struggle for patients.

“I’d like to do it more often but that isn’t possible. Domestic help, for instance…terrible. But on the other hand I also think: my house is clean. Everything spic and span. But it vexes me, that I can’t do it myself.” (ID 12)

## Discussion

Physical health and social health emerged as important overarching themes, while domains associated with mental health were hardly chosen or mentioned. Physical health emerged as the most important theme from the spontaneous statements that patients made when asked about HRQoL in relation to COPD, while two of the six most frequently chosen PROMIS domains (fatigue and physical functioning) pertain to the overarching domain of physical health [40]. Coping with COPD emerged as the second most important theme from the spontaneous statements, while the remaining four PROMIS domains pertained to social health (instrumental support, ability to participate in social roles and activities, companionship, and emotional support) [40]. In addition to the obvious overlap between the outcomes of the open question and the card sorting task (PROMIS domains) with respect to physical health, there is a more subtle overlap with respect to coping. Analysis of the statements patients made to explain their choice of PROMIS domains, showed that coping was a frequently mentioned theme for three of the six most popular PROMIS domains. It should be noted that the statements elicited by the cues (PROMIS domains) were far richer in content than the spontaneous statements, providing us with a lot of information on the patients’ perspectives on these different domains of HRQoL. However, the open question elicited highly valuable information as well, since patients were free to describe HRQoL in their own words; this resulted in an important main theme (coping with COPD) that is not explicitly covered by any of the existing PROMIS domains. These findings underline the importance of using a funnel-shaped questioning strategy, as recommended by Brod and colleagues [27].

Surprisingly, very few patients mentioned or chose domains pertaining to mental health; only two patients mentioned anxiety when answering the open question, while one patient mentioned anger. During the card sorting task, four patients chose the PROMIS domain anxiety, and two patients chose anger. The PROMIS domain depression was not chosen by a single patient. These findings are striking, since the literature indicates that the prevalence of depression or the level of depressive symptoms is much higher in patients with COPD than in the general population; and also anxiety has been shown to be a serious problem in COPD [41]-[46]. Depression is still a taboo-subject for many; especially elderly people have been found to underreport depressive symptoms [47]. It should be noted, though, that although depression was not explicitly chosen, depressive feelings were mentioned by some patients in relation to fatigue, autonomy and acute exacerbations. The interviewed patients may have felt a bit daunted when confronted with the word directly. Additionally, many COPD patients are plagued by feelings of guilt [48], knowing that they are at least partly responsible for the onset of their disease (tobacco smoking). As a result they may feel the need to keep a stiff upper lip, believing they have no ‘right’ to complain, resulting in an underreporting of psychological distress. We discussed this point with a number of COPD experts (researchers and pulmonologists), and several of them pointed out that it requires quite some probing in an interview/consultation to get the patients to open up about their depressive feelings. We therefore presume that the patients not choosing depression may have been a method-effect, and it cannot be ruled out that this domain is in fact relevant to patients with COPD. When it comes to anxiety, Strang and colleagues [46] found that patients experienced high levels of anxiety related to COPD symptoms, mostly in connection with the themes death anxiety (fear of suffocation, awareness of death, fear of dying, separation anxiety) and life anxiety (fear of living, fear of the future). Furthermore, patients also talked about coping strategies they used to deal with the feeling of suffocation, or fear of dying. In their study, Strang and colleagues started with a very general question (“Can you describe what living with COPD means to you?”), but continued with probing questions, including “What are your experiences of anxiety?” Their asking about anxiety directly may partly explain the differences between the results of our open question and their findings. When comparing these anxiety-related themes to the PROMIS anxiety item bank, it becomes apparent that the PROMIS anxiety items are formulated in a very general way. This may be an explanation as to why only few patients in this study chose this domain. It may make more sense to include disease-specific anxiety items that are directly related to COPD symptoms (such as shortness of breath) in an instrument that aims to measure HRQoL in COPD patients, rather than using the PROMIS anxiety domain.

When comparing the most important themes and domains that emerged in this study, to domains covered by the most frequently used instruments to assess HRQoL in COPD [5], a few things stand out. Among the domains most frequently covered by existing instruments are energy/fatigue and social functioning; our findings clearly show support for these being important domains from the patient perspective. However, although many existing instruments focus at least in part on COPD symptoms (dyspnea, cough, phlegm, chest tightness), only few focus on the impact of COPD on daily life [5]. Our results show that this is an important shortcoming of the existing instruments, as many patients indicated they struggle with the impact of COPD on daily activities, as well as finding a way to cope with this negative impact. Most patients indicated that physical activity triggers or aggravates their symptoms, and several patients indicated that they have to avoid or adapt their daily activities. For some patients, these limitations were so severe that they considered themselves as invalids. Many patients struggle daily with their dependency on others in performing daily life tasks; they feel ashamed and frustrated that they are no longer self-reliant.

It should be noted that some patients found it difficult or refused to follow the instructions. Four patients did not pick any PROMIS domain. They argued that they were in good physical shape or were at least able to do everything they wanted; or they fully accepted the consequences of COPD and were able to adjust to it. It may be that the PROMIS domains were too general, which could be solved by adding a disease-specific module to the PROMIS domain framework. Some patients had comorbid disorders and indicated that it was difficult for them to identify the exact impact of COPD. Other patients attributed part of the symptoms that impacted their HRQoL to the ‘normal’ ageing process. Some patients had trouble to pick exactly five important domains. Patients that picked fewer than five domains indicated that they had trouble seeing the difference among some of the domains.

## Conclusion

The most relevant (sub)domains of HRQoL for patients with COPD were: physical health (fatigue, physical functioning), social health (instrumental support, ability to participate in social roles and activities, companionship, and emotional support), and coping with COPD. The latter is not explicitly covered by any of the existing PROMIS domains, or by the most popular traditional questionnaires that are used to measure HRQoL in COPD. Our results reaffirm what has been pointed out in previous studies [27]: engaging patients in PROM development and evaluation is crucial to ensure content validity. The identified PROMIS domains could form the basis for a CAT measuring HRQoL in COPD patients. We recommend the development of a disease-specific module to be included in the CAT, to capture important HRQoL aspects not covered by the current PROMIS domains. When selecting or developing instruments, we argue that it is important to take into account expert opinion as well as patient experience (see Additional file 1). Therefore, we will conduct interviews with healthcare professionals in a future study. To ensure the cross-cultural relevance and external validity of our findings, we will repeat these interviews (with patients and healthcare professionals) in other countries as well, starting with Canada.

## Competing interests

The authors declare that they have no competing interests.

## Authors’ contributions

MP was involved in the design of the study, analysis and interpretation of data, and drafted the manuscript. CB was involved in the design of the study, critically reviewed all aspects of data collection, analysis and interpretation, and revised the manuscript critically for important intellectual content. LL played a key role in data analysis and the interpretation of the data, and revised the manuscript critically for important intellectual content. LG collected the data, and made a contribution to data analysis and revision of the manuscript. SA and OE drafted Additional file 1 and critically reviewed all aspects of the study, including the manuscript. CT was involved in the design of the study, selected PROMIS example items, and critically reviewed all aspects of the study, including the manuscript. JP was involved in the design of the study and the drafting of the manuscript. All authors read and approved the final manuscript.

## Additional files

## Supplementary Material

Additional file 1: Outlines the framework we use in developing a CAT to measures HRQoL. Click here for file

Additional file 2: Constitutes the interview scheme used to gather data for this study. Click here for file

Additional file 3: Lists the 16 PROMIS domains (with example items) the patients had to choose from during the card sorting task. Click here for file
