# Supplementary material for: Identifying key domains of health-related quality of life for patients with Chronic Obstructive Pulmonary Disease: the patient perspective
Source: Health Qual Life Outcomes. 2014 Jul 9;12:106. doi: 10.1186/s12955-014-0106-3 (PMC4226986; doi:10.1186/s12955-014-0106-3)
Supplement: Additional file 1: — Outlines the framework we use in developing a CAT to measures HRQoL. [file s12955-014-0106-3-S1.pdf]

Appendix I

The work to develop a COPD item bank will be conducted simultaneously in the Netherlands and Canada. A series of steps adapted from the PROMIS framework will guide and standardize the process that will be used by both groups. The steps include defining the domains and subdomains that will provide a comprehensive assessment of HRQoL among individuals with COPD, creating a potential item pool, testing the psychometric properties for the item pool, developing a CAT version, and ensuring at each step that the domains, subdomains, and items are culturally appropriate. The table below summarizes each of these steps in more detail. Once a CAT version is created, future work will continue to test the reliability, validity and responsiveness of the CAT and short form versions. At each step, both groups will ensure that the same criteria are used to include or remove items. The framework will help ensure that the steps followed in each setting are consistent and will facilitate communication about the results and combined dissemination of the item bank. Joint development of the item bank will provide a common standard to facilitate comparison of COPD PROs in clinical care, research, and population monitoring.

Recommended Step	Examples of input/activity	Product
**Step 1: Define target domains and subdomains**	- COPD patients interviews<br>- COPD clinician interviews<br>- Literature Review, impact of COPD<br>- Wilson and Cleary, and PROMIS Frameworks	A list of target domains, validated and prioritised by patients and clinicians
**Step 2: Compose and/or select individual items for each (sub)domain**	- PROMIS Item Banks<br>- Other Generic Questionnaires<br>- COPD - Specific questionnaires (e.g. SGRQ, CAT, CCQ, McGill COPD Questionnaire)<br>- Cognitive interviewing to review readability, literacy, cultural harmonization and translatability (French and Dutch)	A pool of potential items for each domain/subdomain
**Step 3: Construct item pool for each identified (sub)domain**	- Test that items cover breadth of (sub)domain, match conceptualization	A pool of potential items for each (sub)domain ready for psychometric testing

	of target domain, and ensure adequate coverage for each language	
**Step 4: Determine psychometric properties of item banks**	- Evaluate psychometric characteristics of items in a representative sample of individuals with COPD, and test for potential DIF for key groups (COPD severity, language, etc.)	Calibrated item bank for each (sub)domain
**Step 5: Develop a Multi-dimensional Computerized Adaptive Testing (CAT)**	- Evaluate and compare psychometric properties across different formats and ensure comparability between paper and CAT administrations	Final version of Multi-dimensional Computerized Adaptive Testing of HRQoL

The Wilson & Cleary model provides a theoretical approach to conceptualising health and quality of life that can be used to guide the evaluation of outcomes. Wilson & Cleary divided health outcome into five domains: biological & physiological variables, symptoms status, functional status, general health perception and overall QoL (1).

The PROMIS framework provides a further breakdown of the Physical, Mental, and Social Health domain into additional sub-domains (2).

References

1. Wilson IB, Cleary PD. Linking clinical variables with health-related quality of life. A conceptual model of patient outcomes. JAMA : the journal of the American Medical Association. 1995 Jan 4;273(1):59-65.

2. PROMIS. Domain Frameworks PROMIS Adult Self-Reported Health. 2014 [2/25/2014]; Available from: http://www.nihpromis.org/measures/domainframework1.
