# Supplementary material for: Identifying key domains of health-related quality of life for patients with Chronic Obstructive Pulmonary Disease: the patient perspective
Source: Health Qual Life Outcomes. 2014 Jul 9;12:106. doi: 10.1186/s12955-014-0106-3 (PMC4226986; doi:10.1186/s12955-014-0106-3)
Supplement: Additional file 2: — Constitutes the interview scheme used to gather data for this study. [file s12955-014-0106-3-S2.pdf]

Appendix II

Script and interview scheme “Interviews with COPD patients about quality of life”

Written by Muirne Paap, Christina Bode & Lianne Groen, University of Twente

Translated to English by Mitzi Paap

Interviews with COPD patients about quality of life

Name interviewer:

Name respondent:

Date:

Time:

Place:

1. Introduction

Introducing myself

Welcome. My name is Lianne Groen, and I study psychology at the University of Twente. Currently, I am conducting a research study on health-related quality of life in COPD patients.

Subject

There are many types of questionnaire in existence to assess quality of life in patients with chronic diseases. All these questions can be subdivided into domains. My research aims to assess, by means of interviews, which of these domains are the most relevant in connection to COPD, from the patient's perspective. In this way, a questionnaire may be developed which assesses quality of life in COPD patients better and more efficiently than current questionnaires.

Processing data

The interviews will be analyzed and recorded in a report. Possibly, the report will also be published.

Confidentiality

The research results will be used for scientific purposes only. Your personal information will not be identifiable.

Duration

The interview will take 20-45 minutes.

Audio recording

During the interview I will take notes. In addition, I would like to audio record the interview, as it will improve the accuracy of my report. Please, be assured that the audio recordings will be deleted when the research has been completed.

Do you object to the use of an audio recorder during the interview? YES/NO

Any questions or remarks at this stage?

Before we start, I would like to ask you to carefully read this consent form. I want to be certain that you are well informed of the research study. I want to stress that it is not a contract, and that you may withdraw from the study at any time, without giving reasons. Ceasing your participation in the research study will have no consequences for your treatment. By signing this form, you indicate that you have read the aforementioned information and that you agree to the conditions.

-Present form, switch on audio recorder-

2. Central focus

We shall now start the interview. Remember that there are no right or wrong answers. It is about your personal situation and your personal experience. Take your time before answering a question. Let me know if you need a break.

-start audio recording-

2.1. General questions

I would like to begin with a few general questions and questions about your illness.

- **GOLD classification** (obtained from the pulmonologist)**:**

- **Gender:** MALE/FEMALE
- *May I write down your date of birth?*

2.2. Roles

I would like to ask a few questions concerning daily activities. These questions are part of an existing questionnaire and may not necessarily apply to you.

Activity	***yes/no***	***Comments***
Are you currently employed?		
Do you perform certain household chores?		
Are you caring for any household members? (children, partner, etc.)		
Are you caring for any non-household members?		

Do you work as a volunteer?		
Have you got a garden? If so, do you tend it yourself?		
Have you got any pets?		

2.3. <u>Questions related to COPD</u>

- *Since when have you received treatment for your COPD?*

- *Have you ever been hospitalized for your COPD?* YES/NO (**If YES**: *How often?)*

- *How often did you have a COPD exacerbation over the last year?*[1]

- *Have you got other illnesses or complaints besides COPD?* YES/NO

2.4. <u>Domains</u>

2.4.1. Open question

We now have come to the part that deals with the different domains of quality of life. I would like to begin with the following question:

- *How does COPD affect your quality of life?*

[1] *COPD exacerbation, flare-up, or lung attack:* a short period during which complaints such as an increase in shortness of breath and an increase in the amount of phlegm (mucus) occur, which necessitate the use of corticosteroid pills (prednisone) or antibiotics.

2.4.2. Cards with domains

I will put down sixteen cards in front of you. On these cards, sixteen domains of quality of life are described, each accompanied by a few examples. I would like you to choose five domains of quality of life which you feel are the most affected by your COPD. Try not to focus on the example questions, as you don't need to answer them. They only indicate what the theme signifies. Concentrate instead on the general contents of each domain.

-let the respondent choose five cards-

You indicate that the following five domains are affected the most by your COPD:

-read the texts on the five cards aloud-

Carefully study these cards again. Arrange the cards from least important to most important.

-let the respondent arrange the five cards-

The way you have arranged the cards indicates that the following domains are the most relevant in connection with your COPD:

1.
2.
3.
4.
5.

-Per domain-:

- *Can you indicate why this domain is important with regard to your COPD?*

1.

2.

3.

4.

5.

3. **Conclusion:**

In this research study you have indicated that **-five domains-** *these are the five domains that are the most affected by your COPD. You point out that:* **-give reasons- (also the ones mentioned before!)**

- *Did I summarize it correctly?* YES/NO
- *Have you got any additional comments?*

- *This is the end of the interview. Have you got any questions or remarks?*

- *How do you feel about this audio interview?*

Thank you for your time and cooperation.

-stop audio recording-
