# Supplementary material for: Identifying key domains of health-related quality of life for patients with Chronic Obstructive Pulmonary Disease: the patient perspective
Source: Health Qual Life Outcomes. 2014 Jul 9;12:106. doi: 10.1186/s12955-014-0106-3 (PMC4226986; doi:10.1186/s12955-014-0106-3)
Supplement: Additional file 3: — Lists the 16 PROMIS domains (with example items) the patients had to choose from during the card sorting task. [file s12955-014-0106-3-S3.pdf]

Appendix III: Domain cards with example items.

**Domain**		Example 1	Example 2	Example 3
**Anxiety**	The past 7 days:	I felt anxious	I found it hard to focus on anything other than my anxiety	I had sudden feelings of panic
**Depression**	The past 7 days:	I felt upset for no reason	I felt depressed	I felt unhappy
**Fatigue**	The past 7 days:	How often did you have to push yourself to get things done because of your fatigue?	How often did your fatigue make it difficult to plan activities ahead of time?	To what degree did you feel tired even when you hadn't done anything?
**Physical function**	With how much difficulty:	Are you able to put on a pullover sweater?	Are you able to run 100 yards (100 m)?	Are you able to turn from side to side in bed?
**Anger**	The past 7 days:	I was irritated more than people knew	I felt envious of others	I felt like I was ready to explode
**Companionship**	To what extent:	Do you have someone with whom to relax?	Can you find companionship when you want it?	Do you have someone with whom to have fun?
**Sleep related impairment**	The past 7 days:	I had a hard time getting things done because I was sleepy	I still felt sleepy when I woke up.	I had a hard time concentrating because I was sleepy
**Pain interference**	The past 7 days:	How much did pain interfere with your close personal relationships?	How much did pain interfere with your enjoyment of social activities?	How often was your pain so severe you could think of nothing else?
**Pain behavior**	The past 7 days	When I was in pain I grimaced	I had pain so bad it made me cry	When I was in pain I screamed
**Satisfaction with participation in social roles and activities**	To what extent:	Are you satisfied with your ability to do household chores/tasks?	Are your satisfied with your ability to do all of the leisure activities	Are you satisfied with your ability to run errands?

			that are really important to you?	
**Ability to participate in social roles and activities**	To what extent:	Do you have to limit the things you do for fun with others?	Do you have trouble doing everything for your friends that you feel you should do?	Do you have trouble doing all of your regular leisure activities with others?
**Sleep disturbance**	The past 7 days:	I tossed and turned at night	I got enough sleep	I had trouble stopping my thoughts at bedtime
**Informational support**	To what extent:	Do you have someone to turn to for suggestions about how to deal with a problem?	Do you get useful advice about important things in life?	Do your friends have useful information to help you with your problems?
**Instrumental Support**	To what extent:	Do you have someone to take you to the doctor if you need it?	Do you have someone to help you clean up around the home if you need it?	Do you have someone to pick up a prescription if you need it?
**Social isolation**	To what extent:	Do you feel that people avoid talking to you?	Do you feel isolated from others?	Do you feel that some of your friends avoid you?
**Emotional support**	To what extent:	Do you have someone to confide in or talk to about yourself or your problems?	Do you feel there are people who really understand you?	Do you have someone who makes you feel needed?
